# Diagnostic and Practical Essays on Medical and Surgical Subjects, Illustrated by Cases

**Published:** 1836-10

**Authors:** 


Art. XII.—*Diagnostisch-praktische Abhandlungen aus dem Gebiete der Medicin und Chirurgie durch Krankheitsfälle erläutert.* Vom Dr. Löwenhardt. *Erster Theil.—Prenzlau*, 1835. 8vo. pp. 352.

*Diagnostic and Practical Essays on Medical and Surgical Subjects, illustrated by Cases.* By Dr. Löwenhardt. *Part First.—Prenzlau*, 1835.

This work consists of three essays,—1. On the Pathognomonic Signs of Diseases, especially Pneumonia; 2. On the Typhoid Symptoms connected with Scarlatina; 3. On Acute and Chronic Inflammation of the Ovary, or Oophoritis. Of the first two we merely mention the titles, as not containing anything peculiarly new or interesting: of the third essay we must speak more fully.

Oophoritis, or inflammation of the ovary, is a disease little known to practitioners as a distinct affection; not so much on account of its rarity as from the obscurity of its symptoms. Dr. Löwenhardt's observations on this affection are practically valuable and deserve attention. Dr. L. considers that examination per rectum is almost the only certain mode of detecting this disease; that, on account of the situation of the ovaries, it is impossible to gain the necessary information by examination per vaginam: whereas, per rectum, the finger can reach the affected part, and detect the existence of tenderness on pressure; and, if the ovaria be at all enlarged, which is usually the case, the gland itself can be distinctly felt.

There seem to be two periods in a woman's life at which the ovaries are more liable to become inflamed than at any other: the one is shortly before, during, and immediately after the appearance of the menses; the other, shortly after labour or premature expulsion, where the increased activity of the circulation, which had been directed towards the uterus, is now spent on other and adjacent organs.

The symptoms are not always precisely the same, being considerably modified according to the different organs which are affected at the same time; and these will very much depend upon which side of the ovary is affected. It is an established practical fact, that acute inflammation of deep-seated parts, or of the ovary itself, especially after labour, seldom exists without spreading to the neighbouring organs: it may remain distinct for a short time, but, as the disease advances, the peritoneum soon becomes affected: hence it is of great importance to form our diagnosis as early as possible.

"As long as the inflammation is confined to the ovarium itself, the seat of the disease can only be shown by the pain, since there is no functional disturbance to mark its presence. Immediately over the symphysis pubis on the affected side, (both ovaries are seldom inflamed at once,) between the groin and the uterus, the abdomen is painful and somewhat tense; at times it is distinctly swollen, and hotter than natural. The pain is seldom violent, rather dull, but becomes sharper and darting as soon as the peritoneum is involved; the part is painful on pressure, and on suddenly assuming the erect posture; and, as long as the inflammation does not spread, remains confined to the affected spot. Usually, however, the inflammatory process rapidly extends at an early period to the peritoneum, especially when under circumstances which predispose this membrane to inflammation, viz. the puerperal state, and, besides the sharp darting pain above mentioned, produces affections either of the bladder or the rectum. In the former case, patients complain of frequent desire to pass water, and scalding even to a painful degree when evacuating the bladder, so as to be easily mistaken for inflammation of its mucous lining; the neighbourhood of the bladder is felt tense, and is very tender on pressure. The urine also is mostly high-coloured, and is passed in the usual quantity in spite of frequent interruptions. The function of the rectum is but little impeded. On the other hand, when the irritation has spread to the posterior portion of the peritoneum, the characters of the disease are very different; the bladder now is less affected than the rectum. In this case, the patient has a sensation of painful pressure in the cavity of the pelvis, amounting to bearing down; the hypogastric region is not so tense or hot, and less sensitive to external pressure. Fruitless forcing to evacuate the bowels arises, frequently amounting to actual tenesmus." (P. 306.)

Without the aid of examination per rectum, it would be exceedingly difficult to form a certain diagnosis: the finger, per anum, easily reaches to the side of the uterus, where the swollen and generally painful ovary may be distinctly felt. Examination per vaginam leads to little or no certain results. We have, it is true, a number of indistinctly marked symptoms, which show that inflammatory action is going on. The vagina is warmer than natural; the os and cervix uteri are neither painful nor swollen at the beginning of the disease: in some cases there is a slight degree of tumefaction of this part, such as is observed shortly after conception. The constitutional symptoms differ but little from those which accompany other inflammatory affections of the pelvic organs, and, as long as the ovary alone is affected, are not of any great extent.

The celebrated Carus of Dresden was the first who made the assertion that the proximate cause of nymphomania depended on an inflamed state of the ovaries, on the ground that these organs are the chief seat of venereal pleasure in the female. This view has been adopted by various continental authors, without attempting in the slightest degree to ascertain its correctness. We have elsewhere and long since expressed our doubt as to the accuracy of Professor Carus's opinions on this point, and we find this doubt confirmed by the author. We do not deny the possibility of ovarian disease resulting from a state of constant and salacious excitement, (the same is known to occur with the male testis;) but, that an inflammatory state of the ovaries is the necessary condition to nymphomania, we much question. We have never yet seen a case arising from this cause; whereas, we have frequently witnessed cases of considerable venereal excitement arising from an inflamed condition of the vagina and external parts. On the other hand, inflammation of the ovary decidedly occurs, not only without the slightest approach to nymphomania, but is frequently attended by a directly opposite state of feeling on the part of the patient. In a patient at this moment under our care, where the left ovary can be distinctly felt per rectum, enlarged, hard, and painful, this is especially the case; sexual intercourse being not only a subject of dread, but even aversion. Dr. Lowenhardt has devoted several pages to disproving these views.

In order to give our readers a better view of the character and phænomena of this disease, we will briefly quote his 4th Observation, which is rendered more interesting from the patient's having had a relapse of her complaint.

"Mrs. S—, æt. 40, of middling stature, delicate figure, and florid complexion, mother of several children, (the youngest of which is eight years of age,) having hitherto enjoyed good health, was attacked on March 12th, 1829, with pains in the abdomen, when the catamenial period was just over, in consequence, as she supposed, of catching cold: these pains increased considerably the following day, and compelled her to keep in bed. She complained of a continued throbbing pain on the right side of the abdomen, in the ovarian region, and a violent desire to pass water, accompanied with much painful scalding: the urine red and clear. On closer examination, the abdomen appeared nowhere enlarged or tender, except in the above-mentioned spot, which was somewhat swollen; and pressure here increased the pain considerably. The vagina was hot, but not painful, neither was the rectum; but, upon examination with the finger through this passage, the ovary of the right side of the uterus was found swollen and painful. There was general constitutional suffering; the patient was feverish, with thirst, flushed cheeks, suffused eyes, a white dry tongue, pain of head, pulse quick, but neither full nor hard. She was put on a strict antiphlogistic treatment, and recovered in the course of eight days.

"On the 17th of April of the following year, an alarm of fire in the night was the cause of her catching another severe cold. She passed a sleepless night, had frequent rigors, with pain in the same side of the abdomen as in the former year, and suppression of the catamenia, which happened to be then present. The next morning she complained of dull pain on the right side of the abdomen, in the same spot as formerly, much increased on pressure; but it appeared to be deeper seated this time, and the abdomen was not so swollen. She experienced a constant forcing to evacuate the bowels without effect, but this time she had no difficulty in passing water. The catamenia had ceased entirely, and the vagina felt hot and dry. Introduction of the finger into the rectum produced pain. The ovary was evidently in a state of inflammation, but this time it was more swollen and painful. The constitutional symptoms were more marked during this attack: the skin was hot and dry, and she had much thirst. She complained that her head was confused; the pulse was 126, not particularly hard; the urine sparing and red. She was bled to ten ounces; twelve leeches were applied to the abdomen, which was afterwards fomented with a narcotic application; and she took a grain of calomel every two hours.

"19th. Her general condition appeared somewhat improved, but the pain of the abdomen was not abated, and the impulse to strain (during which only a small quantity of mucus passed,) was rather increased. The bowels had not been moved, although she had taken ten grains of calomel, and enemata had been instantly returned without effect. Twenty more leeches were applied to the painful spot, and, besides the calomel powders, she was directed to take an oleaginous emulsion.

"20th. The bowels acted twice during the night, and the irritable state of the rectum was somewhat diminished, but the pain in abdomen was not much abated; the pulse continued quick, although neither full nor hard; the heat of surface was moderate; urine red and thick. Ten more leeches were applied. She was directed to rub in a drachm of mercurial ointment every two hours, and take a warm bath.

"22d. The night was passed more quietly than hitherto; the symptoms were diminished. The same remedies were again given at longer intervals, and the warm bath again ordered.

"23d. After a restless night, the local and general symptoms were found again aggravated. Twelve ounces of blood were taken, in spite of her apparent debility. On tying up her arm, she fainted. In order to modify the action of the bowels, which had been much increased by the calomel, I added a little ext. opii to the emulsion, and stopped the mercurial frictions.

"This last bleeding produced a complete change. The next morning, every feeling of pain had nearly ceased; the action of the mercury began to show itself upon the gums and salivary glands. Her recovery was somewhat retarded, from the nurse's having continued the mercurial frictions the following night, contrary to order." (P. 338.)

The essay concludes with a short chapter on Chronic Oophoritis, "for which the texture of the ovaries seems to afford ample occasion:" it is indicated by almost precisely the same symptoms as the acute, only in a less severe degree, and with a less rapid course.

We consider Dr. Lowenhardt's little work as very creditable to the industry and calm practical spirit of the author, and shall be glad to see the continuation of it.

